# Effect of Crystallization on Shape Memory Effect of Poly(lactic Acid)

**DOI:** 10.3390/polym14081569

**Published:** 2022-04-12

**Authors:** Danli Nie, Xianze Yin, Ziqing Cai, Jintao Wang

**Affiliations:** 1 College of Materials Science and Engineering, Wuhan Textile University, Wuhan 430200, China; niedl_1999@163.com (D.N.); yinxianze@wtu.edu.cn (X.Y.); 2 College of Materials Science and Engineering, Wuhan Institute of Technology, Wuhan 430205, China; 3 College of Chemistry and Chemical Engineering, Ankang University, Ankang 725000, China

**Keywords:** poly(lactic acid), annealing, shape memory, crystallization

## Abstract

The opportunity for the preparation of high-performance shape memory materials was brought about by the excellent mechanical properties of poly(lactic acid) (PLA). As the effect of crystallization on shape memory was still unclear, this brings constraints to the high-performance design of PLA. The PLA plates with different aggregation structure were prepared by three kinds of molding methods in this paper. The PLA plates were pre-stretched with a series of different strains above glass transition temperature (i.e., 70 °C). The recovery stress and ratio of the material were measured above stretching temperature (i.e., 80 °C). Prolonging of annealing time resulted in more perfect crystal structure and higher crystallinity. The crystal region acted as network nodes in shape memory PLA, and crystal region structure determined the shape memory performance. Based on the experimental results, the structural evolution of network nodes in shape memory PLA was established.

## 1. Introduction

Shape memory effect is a phenomenon in which the designed shape can recover under external stimulation [1]. It widely exists in metal materials and polymers [2,3,4]. It can be divided into reversible and irreversible shape recovery modes. There is a great difference between metal and polymer materials in terms of shape memory effect [5,6,7]. The recovery stress of metal-based materials is much higher than that of traditional polymer materials [8,9]. Its excellent shape memory properties make it appropriate to be widely used in biomedical and aerospace fields. Therefore, large recovery stress is one of the breakthrough directions of polymer-based memory materials.

There are many kinds of traditional shape memory polymers, including polyurethane, polycaprolactone, styrene-butadiene-styrene, poly(lactic acid) [10,11,12,13,14]. Among them, shape memory materials represented by polyurethane have been studied deeply and systematically [15,16,17,18]. Polyurethane shape memory products have been widely used in textile, medical, intelligent manufacturing, and other fields. However, some special properties of matrix are required in some fields [19]. For example, screws and nuts used in surgery need to be of high strength, biocompatible, and biodegradable. They do not need to be removed after surgery, which can greatly reduce the patient’s pain. In space exploration, the use of degradable polymer materials can provide a feasible way to solve space debris [20,21].

PLA is obtained by the polymerization of lactic acid monomer, and it is common in PLLA products. It has excellent mechanical properties, biocompatibility, and degradability [22]. Under the stricter international environmental protection policies, modified and functional PLA products are more and more widely used. However, this material also has obvious brittle characteristics, which brings difficulties to the later structural design. In most cases, toughening modification needs to be applied to PLA matrix first [23,24,25]. In conclusion, toughening modification can significantly improve the flexibility of PLA, while it results in the obvious decline of mechanical performance.

How to combine its excellent mechanical properties and improve shape memory performance of PLA is the focus of this paper. In particular, PLA is a semi-crystalline polymer and the roles of crystal and amorphous regions in shape memory models are ambiguous. In the classical polymer shape memory system, the network nodes are usually composed of covalent bonds, which has higher thermodynamic stability. After repeated tensile deformation, it can maintain good recovery characteristics. Therefore, efficient construction of network nodes with higher thermal stability is the key to improve shape memory performance. This is also the focus of the PLA system research.

In this paper, the research on shape memory effect of PLA from the perspective of polymer crystallization is the innovation of this paper. The in-depth analysis of this system can lay a foundation for the functional design of semi-crystalline polymers. The effects of stretching temperature and annealing time on the shape memory effect were investigated. The important role of crystal region in shape memory PLA was emphatically discussed. The evolution of network nodes and memory switches were clarified during deformation, and the shape memory structure model of PLA was established.

## 2. Experimental

### 2.1. Materials

Poly(lactic acid) granule (6202D, *MI* = 15~30 g/10 min) were supplied by NatureWorks, Minneapolis, MN, USA.

### 2.2. Preparation of PLA Plates

The PLA granules were vacuum-dried at 80 °C for 5 h. Then the PLA plates were prepared by compression molding process on a plate vulcanizing machine. The temperature was 185 °C, and the pressure was 10 Mpa. The hot compressing time lasted for 3 min. After the hot compressing was completed, the solidification process can be changed, which can be divided into the following three types:

(1) Cold compression process: The hot mold was placed in another plate vulcanizing machine. The cold compressing temperature was 25 °C, and pressure was 5 Mpa. The entire cool pressing process lasted for 5 min;
(2) Quenching process: After the hot compressing process was completed, the PLA plate coated with aluminum foil was placed in water for rapid cooling. The water temperature was 25 °C;
(3) Annealing process: After the completion of cold pressing process, the PLA plate was placed in a high temperature oven. The thermal treatment temperature was 115 °C, and annealing time lasted for 5 min.

Finally, the prepared PLA plates were cut into standard dumbbell specimens. The length, width and height of the specimens were 45 mm, 4 mm, and 0.4 mm, respectively.

### 2.3. Performance Test and Structure Analysis

The study of shape memory effect of materials was carried out on the tensile testing machine equipped with a high temperature oven. The experimental procedure was divided into three parts: (1) The specimen was stretched to the target deformation $\varepsilon_{m}$ at a rate of 50 mm/min at the set temperature; (2) the specimen was kept in the deformed state and removed from the fixture after it cooled to room temperature; (3) the specimen was heated to 80 °C and kept for 5 min to ensure that the shape no longer returned, and the recovered length of the specimen was recorded as $\varepsilon_{p}$. The following formula was used to calculate the shape recovery ratio (SR):

$$\mathrm{SR}=\frac{\varepsilon_{m}-\varepsilon_{p}}{\varepsilon_{m}}\times 100\%.$$

The test procedure for the measurement of shape memory stress was similar to (1) and (2) above. The specimen was heated to 80 °C and the maximum tensile stress was recorded.

Differential Scanning calorimeter (204 F1, NETCSCH): Thermal analysis of the specimens was carried out under the protection of argon atmosphere. The specimens were heated from 25 °C to 180 °C at the rate of 10 °C/min, and the heating process was held at 180 °C for 2 min. Subsequently, the specimens were cooled from 180 °C to 25 °C at the same rate. The melting point (T_m_), cold crystallization temperature (T_cc_) and glass transition temperature (T_g_) could be obtained by thermal analysis curves.

One-dimensional wide-angle X-ray diffraction (MiniFlex 600, Regaku): X-ray wavelength of copper target was 0.154 nm, the scanning angle range was from 5° to 45°, and the scanning speed was 2°/min. The diffraction spectrum was processed by peak splitting software to calculate the crystallinity. The grain size was calculated by Scherrer formula as follows [26]:

$$D=\frac{K\gamma }{\beta \cos\theta }$$

K is the Scherrer constant, and D is the average thickness of the grain perpendicular to crystal plane. β is the half-height width of the diffraction peak, θ is the diffraction angle, and γ is the X-ray wavelength.

The the degree of crystallinity was calculated by the following equation:

$$\mathrm{Xc}=\frac{\mathrm{Ac}}{\mathrm{Ac}+\mathrm{Aa}},$$

where Xc is the crystallinity, Ac is the area of crystal region based on peak separation processing of WAXD files, and Aa is the area of amorphous region.

## 3. Results and Discussion

### 3.1. Thermal Analysis of PLA Plates

**Figure 1:**
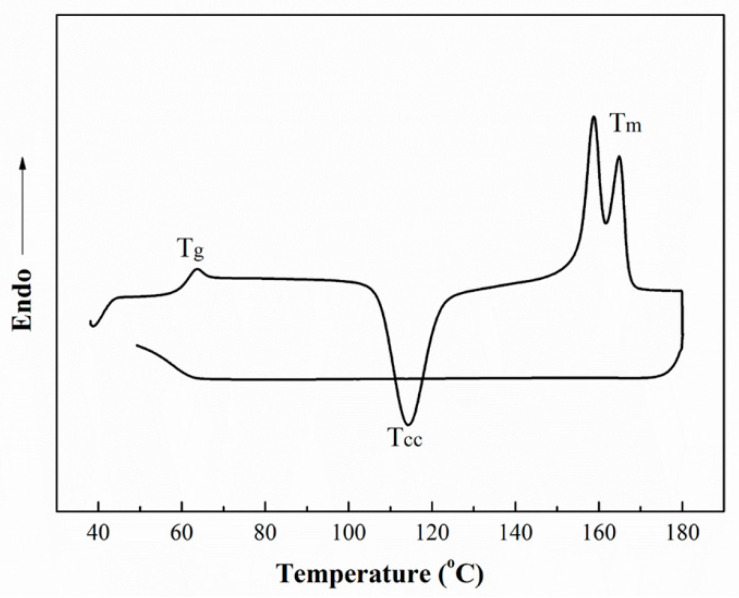
DSC curves of PLA plate prepared by cool compression process.

Figure 1 shows the heating and cooling curves of PLA plate. According to analysis of the DSC curves, the parameters of thermal properties are shown in Table 1. The glass transition temperature (T_g_) and cold crystallization temperature (T_cc_) of PLA are 61.3 °C and 114.3 °C, respectively. This specimen exhibits the characteristics of double melting peaks during the heating process. The melting temperatures are 158.8 °C and 164.8 °C, respectively. Generally, PLA crystallizes poorly. When the heat treatment temperature exceeds the glass transition temperature (T_g_), an obvious recrystallization behavior is observed, which is called cold crystallization. The appearance of double molten peaks is ascribed to the grains with different thermal stability in the PLA matrix [27]. This phenomenon is directly related to the crystallization characteristics of PLA. There is no crystallization peak in the cooling curve. It indicates that crystallization rate of PLA is slow and crystallization behavior cannot be completed in a limited time range.

### 3.2. Shape Memory Performance of PLA Plates

**Figure 2:**
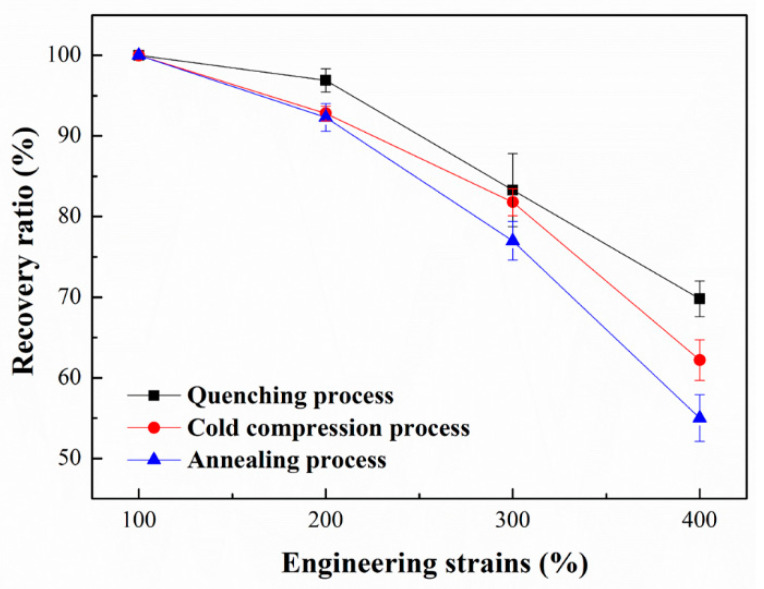
The recovery ratio as a function of pre-stretching strains of three kinds of PLA plates.

Figure 2 shows the recovery ratio of three kinds of PLA plates measured at 80 °C. It can be seen that the recovery ratio of deformation is inversely proportional to the stretching strains. In the shape memory PLA, the crystal region and glass transition temperature (T_g_) serve as physical crosslinking node and shape memory switch respectively [28]. At higher temperatures (i.e., 70 °C), lamellar slippage, fragmentation, and recrystallization tend to occur [29,30]. The crystal destruction process leads to the destruction of the original physical crosslinking points. Moreover, the new network nodes will be formed during the recrystallization process. The entanglement points between molecular chains in the amorphous region will be changed, which destroying the existing shape memory structure. Therefore, the recovery ratio of deformation decreases gradually with the increasing stretching strains.

The annealing time is the main difference of PLA plates prepared by three kinds of molding processes. Cold compression process refers to the slow cooling on the machine at 25 °C. The quenching process refers to rapid cooling at 25 °C in water. Furthermore, the annealing process refers to supererogatory heat treatment at 105 °C for 10 min. In general, these three forming processes cause the difference of annealing time. From the analysis in Figure 2, it can be seen that the recovery ratio of deformation is basically inversely proportional to annealing time. As the heat treatment process directly affects the crystallization properties of PLA, the relationship between crystallization properties and shape memory needs further analysis.

**Figure 3:**
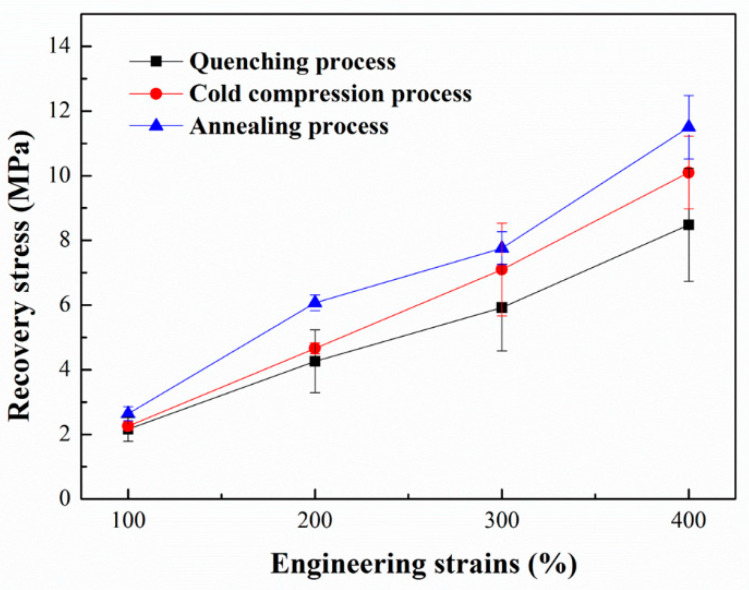
The recovery stress as a function of pre-stretching strains of three kinds of PLA plates.

The recovery stress of the three kinds of poly(lactic acid) plates is shown in Figure 3. With the increase of stretching strain, the recovery stress of the specimens increases. When the memory switch is turned on, more molecular chains relax along the orientation and undergo conformation adjustment, which results in greater recovery stress. Based on comparison of the three forming processes, it can be found that the recovery stress of the samples increases with the extension of annealing time. The annealing process also leads to the perfection of crystal structure and the increase of crystallinity. In PLA memory system, crystal region acts as physical crosslinking nodes. The more perfect crystal structure will results in the more stable physical crosslinked nodes. The higher crystallinity will lead to the more physical crosslinked nodes. This more complete memory system is beneficial to form recovery stress after deformation. This point will be discussed further below.

### 3.3. Crystal Structure Analysis of Polylactide Plate

**Figure 4:**
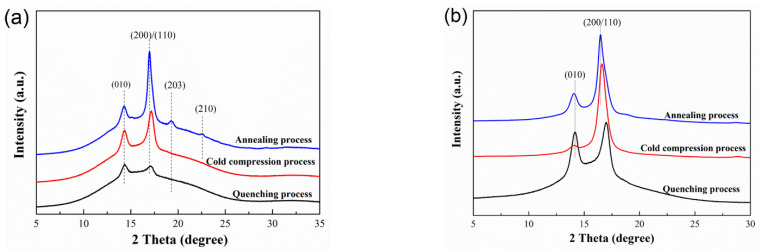
1D-WAXD files of three kinds of PLA specimens: (**a**) non-stretched; (**b**) stretched at 70 °C with 300% strain.

The one-dimensional wide-angle X-ray diffraction files of PLA plates are shown in Figure 4. For non-stretched plate, 2θ = 14.3° and 17.1° corresponds to the diffraction peaks of (010) and (200/110) lattice planes [31,32]. With the extension of annealing time, the diffraction intensity of (010) and (200/110) lattice planes increases gradually. In particular, the diffraction peaks of (203) and (210) planes appear in PLA plate prepared by annealing process. For the stretched plates, two obvious diffraction peaks appear at 14.1° and 16.6°. It can be found that the diffraction peaks move to a lower diffraction angle after stretching. The PLA plates crystallize into α’-form crystal after stretching, and the crystal structure does not change during heat treatment process. After stretching, the diffraction intensity of (200/110) plane is obviously enhanced, indicating that the crystallinity of the matrix is significantly increased.

The half width and relative area of the characteristic peaks can be obtained by peak division of one-dimensional WAXD files. According to these parameters, the values of crystallinity and grain size can be calculated by relevant formulas, and the results are shown in Table 2. With the extension of annealing time, the crystallinity and grain size of PLA plates increase gradually. After stretching, the crystallinity and grain size of PLA plate increase obviously compared with that of unstretched specimens. For PLA plate prepared by cold compression process, the crystallinity increases from 10.2% to 61.1% and the grain size increases from 9.8 nm to 10.2 nm during the pre-stretching process. Thermal treatment and pre-stretching process are conducive to the formation of more stable and perfect crystal structure.

The crystal region acts as the crosslinking nodes and the glass transition acts as the memory switch. The molecular chain orientation of PLA plate will occur after stretching at different strains. The analysis of DSC results shows that the glass transition temperature (T_g_) is 61.3 °C. The pre-stretching temperature is set at 70 °C, which is beneficial to disentanglement and orientation of molecular chains. 

The change of network nodes during stretching is the key factor which affecting shape memory effect. The variation of recovery ratio and recovery stress has been discussed before. The following discussion will focus on the analysis of change tendency of crystal region as a network node in the molding process.

**Figure 5:**
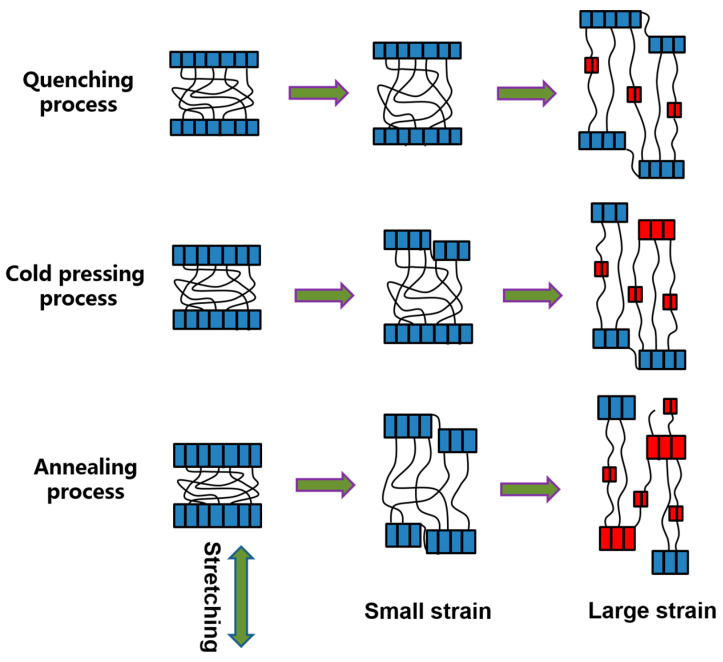
Schematic illustration of structure evolution of three kinds of PLA which stretched at 70 °C.

The structural evolution of the three kinds of PLA plates during stretching process is shown in Figure 5. Blue blocks represent original lamellae and red blocks represent newly generated lamellae. For PLA plate prepared by quenching process, interlamellar slippage occurs at low strain [33,34]. At large strains, intralamellar slippage occurs and orientation induces the formation of new lamellar structures [33,34]. For PLA plate prepared by cold compression process, interlamellar slippage and intralamellar slippage occur at low strains [33,34]. When PLA plates are stretched at large strains, the lamellar destruction (or melting) occurs, and they recrystallize into a new lamellar structure [33,34]. For PLA plate prepared by annealing process, interlamellar slippage and intralamellar slippage are more obvious at low strain. At large strains, obvious lamellar destruction (or melting) occurs and they recrystallized into new lamellar structures. The orientation induced the formation of a large number of small crystals during the stretching process. It can be seen that the crystal structure of PLA is strongly dependent on the molding process and pre-stretching strain. This is also the reason why the shape memory effect of PLA is strongly dependent on molding process.

In combination with the result analysis shown in Figure 2, it can be found that the shape recovery ratio of PLA plate decreases gradually with the increase of stretching strains. When the pre-stretching strain is 100%, the shape recovery ratio reaches to 100%. When the stretching strain is small, the PLA plate tends to experience intralamellar slippage and interlamellar slippage. This structural change does not cause significant changes in network nodes and thus does not restrict the recovery of deformation. When the stretching process occurs at large strains (300% and 400%, etc.), it tends to experience lamellar destruction (or melting) and recrystallization, which results in the formation of new lamellar structures. In this case, the newly formed lamellae are widely distributed in the matrix, thus changing the location and number of network nodes in the memory system. Therefore, when stretched at large strains, the shape of the plate cannot be effectively recovered. Combined with the analysis of the results shown in Figure 2 and Figure 3, it can be found that the shape recovery ratio of PLA plate decreases gradually with the extension of annealing time. Meanwhile, the recovery stress of PLA plate increases gradually. The lamellar size and crystallinity of PLA plate increase after heat treatment. Crystal region plays the role of network node in PLA memory effect system. Stable network nodes are damaged to some extent after stretching. In the annealed PLA specimen, the network nodes are even disconnected from the free molecular chain (Figure 5). And the stretching process can induce the formation of a large number of new network nodes. The formation of these new network nodes changes the movement state of the free molecular chain, which resulting in a lower recovery ratio. Combined with the analysis of the crystalline structure (Figure 4 and Table 2), it can be found that the crystallinity of the oriented PLA plate increases with the extension of annealing time. Therefore, the greater the crystallinity is, the larger the node density of the network is, and the greater the recovery stress is.

## 4. Conclusions

In this paper, PLA plates with structural differentiation were prepared by three kinds of molding process. The shape memory effect of the specimens was measured at 80 °C. With the increase of pre-stretching strain, the shape recovery ratio of PLA decreased gradually, while the recovery stress increased gradually. With the extension of annealing time, the shape recovery ratio of PLA plate decreased gradually, while the recovery stress increased gradually. The stretching process and thermal treatment process were conducive to the formation of perfect crystal structure. In the shape memory system, crystal region acted as network nodes. The memory effect was determined by the change of crystal region content and crystal structure. Higher crystallinity and crystal structure integrity could improve shape memory performance.

## Figures and Tables

**Table 1:** DSC analysis of PLA plate.

Specimen	T_g_	T_cc_	T_m_
PLA plate	61.3	114.3	158.8/164.8

**Table 2:** Crystallization properties of three kinds of PLA specimens.

Specimens	Parameters	Quenching Process	Cold Compression Process	Annealing Process
Non-stretched	Crystallinity (%)	7.7	10.2	13.6
	Crystallite size (nm)	7.7	9.8	12.9
Stretched	Crystallinity (%)	53.4	61.1	69.1
	Crystallite size (nm)	8.5	10.2	13.6

## Data Availability

The data that support the findings of this study are available from the corresponding author upon reasonable request.
